# Chemotherapy in adult patients with pilocytic astrocytoma: a retrospective multicenter cohort study

**DOI:** 10.1007/s11060-025-05275-8

**Published:** 2025-10-15

**Authors:** Laura van den Berg, Joyce Wilbers, Filip Y. F. L. de Vos, Mathilde C. M. van Kouwenhoven, Antoinette Y. N. Schouten-van Meeteren, Johan A. F. Koekkoek, Martinus P. G. Broen, Anja Gijtenbeek, Nathalie E. Synhaeve, Martin J. van den Bent, Jacoline E. C. Bromberg, Marion Smits, Walter Taal

**Affiliations:** 1 Center of Expertise for Cancer Survivorship, Radboud University Medical Center, Nijmegen, The Netherlands; 2 Department of Medical Oncology, Utrecht University Medical Center, Utrecht, The Netherlands; 3 Department of Neurology, Brain Tumor Center Amsterdam, Amsterdam UMC, Vrije Universiteit Amsterdam, Amsterdam, The Netherlands; 4 Princess Máxima Center for Pediatric Oncology, Utrecht, The Netherlands; 5 Department of Neurology, Leiden University Medical Center, Leiden, The Netherlands; 6 Department of Neurology, Haaglanden Medical Center, The Hague, The Netherlands; 7 Department of Neurology, Maastricht University Medical Center, Maastricht, The Netherlands; 8 Department of Neurology, Radboud University Medical Center, Nijmegen, The Netherlands; 9 Department of Neurology, Elisabeth Tweesteden Hospital, Tilburg, The Netherlands; 10 Department of Neurology, Brain Tumor Center, Erasmus MC Cancer Institute, Postbus 2040, Dr. Molewaterplein 40, 3015 GD Rotterdam, Rotterdam, 3000 CA The Netherlands; 11 Department of Radiology & Nuclear Medicine, Erasmus Medical Center, Rotterdam, The Netherlands

**Keywords:** Pilocytic astrocytoma, Adults, Low grade glioma, Chemotherapy, Overall survival (OS), Progression-free survival (PFS)

## Abstract

**Purpose:**

Pilocytic astrocytomas are circumscribed WHO grade I gliomas that predominantly affect children, although they also occur in adults. While maximal safe resection is standard treatment, until recently chemotherapy was often used in pediatrics with irresectable symptomatic tumors to delay radiotherapy. As evidence for this approach in adults is limited, we performed a multicenter retrospective cohort study on this subject.

**Methods:**

Adult patients (≥ 16 years) treated with chemotherapy between 2006 and 2020 across eight Dutch medical centers were included. Treatment response was centrally assessed using RAPNO criteria and survival outcomes were analyzed using the Kaplan–Meier method.

**Results:**

Thirty-one patients with pilocytic astrocytoma were included (median age 27 years). Most patients had received prior treatments, including radiotherapy (*n* = 19) and/or surgery (*n* = 17), with a median interval of over five years from histopathological diagnosis to the start of chemotherapy. Temozolomide was most frequently used (64.5%). The objective response rate for all types of chemotherapy was 42%, with a median progression-free survival of 20 months and a median overall survival of 49 months. Adolescents and young adults (< 40 years) showed significantly better survival outcomes.

**Conclusion:**

Chemotherapy offers meaningful disease control in adolescents and young adults with irresectable symptomatic pilocytic astrocytoma, after prior treatments. However, with the improved efficacy and tolerability of targeted treatments, such as BRAF/MEK inhibitors, treatment is shifting away from traditional chemotherapy and radiotherapy. Nevertheless, chemotherapy may still represent a viable treatment option in adolescent and young adults when no actionable molecular targets are identified or when targeted therapies have failed.

## Introduction

Pilocytic astrocytomas (PA) are circumscribed slow-growing brain tumors, classified as World Health Organization (WHO) grade 1. The development of PA can be secondary to underlying genetical conditions, particularly neurofibromatosis type 1 (NF1) [1]. In rare cases PA show malignant behavior. These more aggressive PA are now largely classified as PA with anaplasia or high-grade astrocytoma with piloid features (HGAP) [2]. Molecularly, PA are characterized by alterations with the BRAF gene, in about 60% of patients with rearrangements (BRAF::KIA fusion). PA with anaplasia and HGAP often show additional alterations in other genes [3–8].

PA are the most common brain tumor in children and young adults and account for 15% of all newly diagnosed central nervous system tumors in this age group. While they can develop at any age, their incidence declines sharply with increasing age [2, 9, 10]. Generally, PA have a favorable prognosis with an overall survival (OS) of above 90% in the pediatric population, but the survival declines with age [2]. This discrepancy most likely reflects the fundamental differences in tumor biology in the pediatric and adults population [11].

Located throughout the neuroaxis, PA are most often located in infratentorial structures (cerebellum, 40%) and midline brain structures (optic pathway, hypothalamus, and brainstem) [10]. The predominant location of NF1 associated PA is the optic pathway (20%). However, only 1 out of the 4 of NF1 associated optic pathway gliomas (OPG) will become symptomatic and seldomly after the age of 7 years. The typical presentation of PA on MRI is a contrast enhancing nodule bounded with a cystic mass [12].

According to European Association for Neuro-Oncology (EANO) and International Society for Pediatric Oncology for Low Grade Glioma (SIOP-LGG) guidelines the primary treatment of PA is maximal safe gross total resection (GTR) [13, 14]. Many patients with PA attain long-term tumor control after GTR. Radiotherapy (RT) or systematic therapy is indicated in case of symptomatic irresectable progressive disease, and provides long term disease control in the majority of patients [15]. Chemotherapy, and more recently targeted therapies, has been proven as an effective therapy in young children with symptomatic irresectable PA and is preferentially used to prevent RT toxicity [16–21].

To our knowledge there is sparse data on chemotherapy in adults with symptomatic irresectable PA. We therefore performed a multicenter retrospective cohort study in adult patients with symptomatic irresectable PA, treated with chemotherapy.

## Materials and methods

### Patients

Data from all patients with histopathologically confirmed PA who received chemotherapy at the age of ≥ 16 years old, between 2006 and 2020, were collected from eight medical centers in The Netherlands. Most tumors were classified as PA according to the WHO 2007 classification. The decision for the start of chemotherapy was made at the discretion of the local treating physician. Patients were required to have a lesion that met the measurability criteria defined by RAPNO [22]. We reviewed their medical records for demographics (age, gender, FN1, histological data, WHO classification, and molecular data), treatment characteristics, and survival. MRI scans were obtained from the period preceding the progression that initiated chemotherapy until further progression on chemotherapy or death. Data was entered into the Castor Database for Electronic Data Capture. Written informed consent was obtained from all patients or, when applicable, from their legal guardians. The Medical Ethics Review Committee (METC) of all participating centers approved this study. This research was conducted in accordance with the Declaration of Helsinki, local, and national regulations.

### Evaluation

Response was assessed with MRI scans made every 3–6 months during chemotherapy and according to local guidelines thereafter. All MRI scans, both baseline and follow-up, were made with and without gadolinium-based contrast agent (GBCA). For tumor measurements the T2 weighted MR images were used for non-enhancing tumors, and GBCA enhanced T1 weighted MR images for enhancing tumors. The response rate was primarily defined using the product of perpendicular diameters of the tumor (and cyst) according to the RAPNO criteria [22]. Responses were categorized as progressive disease (PD), stable disease (SD), minor response (MR), partial response (PR), or complete response (CR). The objective response rate (ORR) included cases classified as CR, PR, or MR [23]. Response was centrally assessed by MS and WT. Chemotherapy-related toxicities were collected according to the NCI Common Terminology Criteria for Adverse Events version 5.0 (CTCAEv5) [24].

### Statistical analysis

The main objectives of this study were to evaluate OS, progression-free survival (PFS), and ORR. The Kaplan-Meier method was used to estimate PFS and OS. PFS and OS were measured in months, from the first day of start of chemotherapy to the date of event, with censoring at the date of last follow-up for survivors. The survival distributions between subgroups were compared using the log-rank test. All reported *p*-values are two sided. In this exploratory analysis no adjustments were made for multiple testing. IBM SPSS statistics version 29 software was used for the statistical analysis.

## Results

### Baseline characteristics

We identified thirty-one patients who had received chemotherapy for PA after the age of 16. Their baseline characteristics are presented in Table 1.

**Table 1:** Baseline characteristics in patients treated with chemotherapy for a pilocytic astrocytoma

Characteristics	No of patients (%)
Total	31
Sex	
Male Female	17 (54.8%) 14 (45.2%)
Age at debut symptoms, median (range)	19 years (3–51 years)
Age at start CTX, median (range)	27 years (16–56 years)
Diagnosis-to-CTX interval, median (range) *	62 months (2–343 months)
Initial symptoms	
Multiple complaints Increased intracranial pressure Visual impairments Cerebellar ataxia Other	16 (51.6%) 4 (12.9%) 5 (16.1%) 1 (3.2%) 5 (16.1%)
Tumor localization	
Brainstem Cerebral Optic pathways Multifocal Cerebellar Spine	8 (25.8%) 5 (16.1%) 6 (19.4%) 7 (22.6%) 3 (9.7%) 2 (6.5%)
Radiological characteristics	
Contrast enhancement Presence of cysts > 3 mm	23 (74.2%) 10 (32.3%)
Pathology	
PA PA with high-grade features*	26 (83.9%) 5 (16.1%)
Molecular tumor characteristics	
Missing data None NF1 mutation BRAF V600E mutation BRAF::KIAA fusion MGMT methylation Chromosomal imbalance**	17 (54.8%) 8 (25.8%) 3 (9.7%) 1 (3.2%) 1 (3.2%) 1 (3.2%) 1 (3.2%)
Diagnosed with neurofibromatosis type 1	
Yes No	5 (16.1%) 26 (83.9%)

CTX: chemotherapy; PA: pilocytic astrocytoma

* PA with high-grade features mostly classified as PA with anaplasia according to the WHO 2017 classification, but could according to the new WHO 2021 classification also include high-grade astrocytoma with piloid features (HGAP)

**Chromosomal imbalance of chromosome 4q, 7, 9 and 12

Just over half of the patients were men, and the median age at the start of chemotherapy was 27 years. Most patients had a WHO grade 1 PA as their primary histological diagnosis, and most tumors were located outside the cerebellum. The median interval from primary histological diagnosis to the start of chemotherapy was 62 months. Excluding biopsies, patients received a median of 1 treatment line (range 0–6) prior to chemotherapy, which is shown in Table 2.

**Table 2:** Treatment before and after the 1st chemotherapy in adult patients with a pilocytic astrocytoma

**Treatment before chemotherapy – median (range)**	1 (0–6)
**Number of patients with treatments - n (%)**	Total (*n* = 31)
No treatments (except biopsies)	6 (19.4%)
Radiotherapy	8 (25.8%)
Surgery	5 (16.1%)
Surgery and Radiotherapy	9 (29.0%)
Surgery and Chemotherapy	1 (3.2%)
Surgery, Radiotherapy and Chemotherapy	2 (6.5%)
**Treatments after chemotherapy – median (range)**	1 (0–6)
**Number of patients with treatments - n (%)**	Total (*n* = 31)
No treatments (except biopsies)	13 (41.9%)
Chemotherapy	5 (16.1%)
Radiotherapy	1 (3.2%)
Radiotherapy and Chemotherapy	2 (6.5%)
Radiotherapy and BRAF/MEK inhibitors	2 (6.5%)
Surgery	2 (6.5%)
Surgery and Radiotherapy	2 (6.5%)
Surgery and Chemotherapy	3 (9.6%)
Surgery, Radiotherapy and Chemotherapy	1 (3.2%)

Patients underwent radiotherapy (*n* = 19) and/or surgery (*n* = 17) before starting chemotherapy. Two patients received gross total resection as first line treatment. Three patients (9.7%) had previously received chemotherapy before the age of 16. Among the 25 patients who received a treatment modality prior to the initiation of chemotherapy, the median interval between the previous treatment modality and the start of chemotherapy was 22 months (range 0–223 months).

### Chemotherapy and toxicity

Most patients (*n* = 20, 64.5%) were treated with temozolomide (TMZ). Five received a combination of procarbazine/lomustine (CCNU) and vincristine (PCV), four with platinum-based chemotherapy (PBC), and two with vinblastine (VBL). Ten patients experienced chemotherapy-related toxicities CTCAEv5 grade 3 or 4, which is shown in Table 3. Eighteen patients (58.1%) completed their chemotherapy treatment regimen.

**Table 3:** Chemotherapy related toxicities in patients treated with chemotherapy for a pilocytic Astrocytoma

CTCAE v5 grade 3 and 4 toxicities	No of patients (%)
No Yes	21 (67.7%) 10 (32.3%)
Bone marrow suppression Carboplatin / procarbazine allergy Fatigue Neuropathy Anorexia Persistent infections Pulmonary complaints Endocrine disorders	4 2 2 2 1 1 1 1

CTCAEv5 = NCI Common Terminology Criteria for Ad5verse Events version 5.0

### PFS, OS, and ORR

The median follow-up from the start of chemotherapy was 44 months (range 2–169 months). Median PFS (mPFS) was 20 months, and median OS (mOS) was 49 months. The 10-year OS was 35%. The ORR based on the RAPNO criteria was 42%, which is shown in Table 4. Patients with SD (48.4%) remained stable for a median of 11 months (range 5–61 months).

**Table 4:** Response rate based on the RAPNO criteria in patients treated with first type of chemotherapy for a pilocytic Astrocytoma

Response no (%)	Total (*n* = 31)	TMZ (*n* = 20)	PCV (*n* = 5)	PBC (*n* = 4)	VBL (*n* = 2)	Prev. RT (*n* = 18)	No prev. RT (*n* = 13)
PR MR SD PD	10 (32.3%) 3 (9.7%) 15 (48.4%) 3 (9.7%)	8 (40%) 3 (15%) 7 (35%) 2 (10%)	2 (40%) 0 (0%) 2 (40%) 1 (20%)	0 (0%) 0 (0%) 4 (100%) 0 (0%)	0 (0%) 0 (0%) 2 (100%) 0 (0%)	8 (44.4%) 2 (11.1%) 7 (38.9%) 1 (5.6%)	2 (15.4%) 1 (7.7%) 8 (61.5%) 2 (15.4%)

RAPNO criteria = Response Assessment for Pediatric Neuro-Oncology criteria, TMZ = temozolomide, PCV = procarbazine, lomustine (CCNU) and vincristine chemotherapy, PBC = platinum-based chemotherapy, VBL = vinblastine chemotherapy, CR = complete response, PR = partial response, MR = minor response, SD = stable disease, PD = progressive disease

**Fig. 1:**
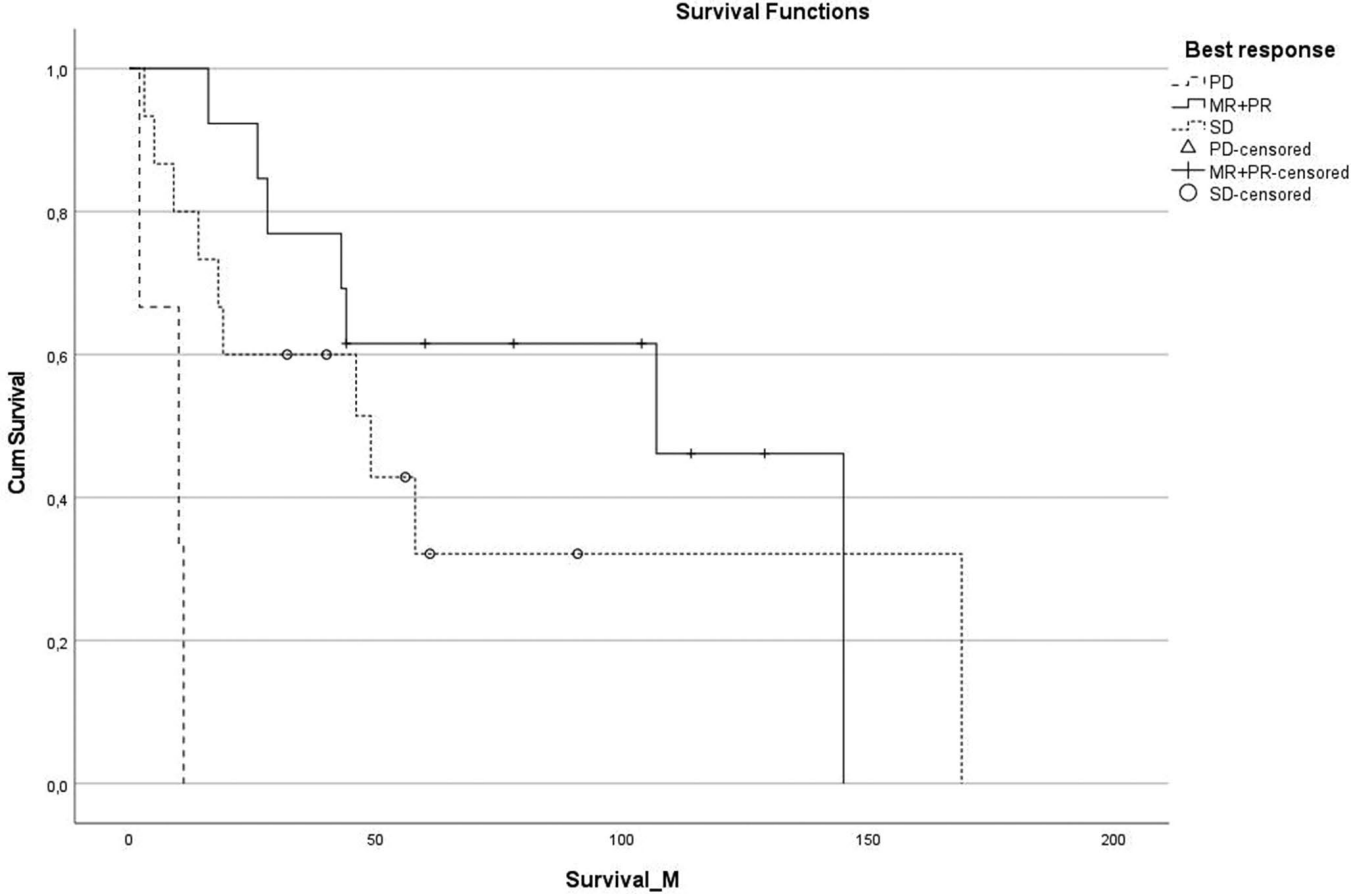
Overall survival since start of the chemotherapy per best response

**Fig. 2:**
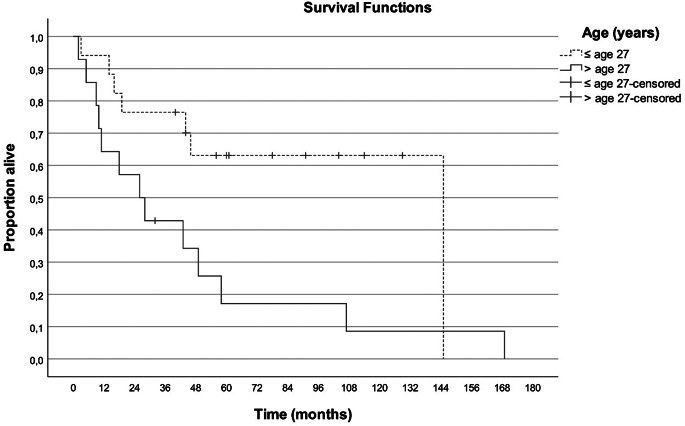
Overall survival since start of the chemotherapy per age group (under or over the median age of 27 years)

The mOS was significantly longer in patients with an objective response (107 months) and SD (49 months) compared to those with PD (10 months) with a *p*-value of < 0.001. The mOS was significantly longer in patients younger than the median age of 27 years (145 months versus 26 months, *p*-value = 0.019). This difference remained significant in the age group < 40 years compared to older patients (107 months versus 26 months, *p*-value = 0.007). The survival curves are shown in Figs. 1 and 2.

There were no significant differences in OS or PFS between patients who were radiotherapy-naive and those who had received radiotherapy prior to chemotherapy, nor between patients who had undergone partial resection and those who had a biopsy. Given the small number of patients with NF1 (*n* = 5), those exhibiting histological high-grade features (*n* = 5), and the limited number treated with specific chemotherapy regimens, it was not possible to perform meaningful comparisons of OS and PFS across these subgroups.

The 3 patients with a NF1 mutation had a clinical diagnosis of NF1: histology showed PA in one patient and PA with anaplasia in two patients. The PA patient received VBL with SD for 7 months and remains alive at 56 months. The 2 PA with anaplasia patients received TMZ, resulting in PR and MR for respectively 17 months and 19 months; the PR patient died at 44 months, while the MR patient is alive at 44 months. The patient with a BRAF V600E mutation received PBC and remains progression-free at 61 months. The patient with a BRAF::KIAA fusion received PCV, had SD for 8 months, and survived 19 months.

### Treatment at progression

Excluding biopsies, patients received a median of one additional treatment modality (range 0–6) following chemotherapy (see Table 2). In cases of disease progression after chemotherapy, subsequent treatments included surgery (*n* = 8), radiotherapy (*n* = 8), BRAF/MEK inhibition (*n* = 2), and/or chemotherapy (*n* = 11). During follow-up, twenty patients passed away, the majority due to complications related to tumor progression. One patient died following surgical complications. No fatalities were attributed to chemotherapy or radiotherapy-related adverse events.

## Discussion

To our knowledge, this multicenter retrospective cohort study is the first to evaluate the outcomes of adult patients with PA treated with chemotherapy. Our results demonstrate that chemotherapy achieved an objective response according to the RAPNO criteria in 42% of patients, with a mPFS of 20 months. Notably, patients who achieved stable disease remained progression-free for a median duration of 11 months. Given that most patients had received prior treatment modalities, these findings suggest that chemotherapy may provide meaningful disease control in a subset of adult patients with symptomatic irresectable PA.

The outcomes observed in our adult cohort contrast notably with those reported in pediatric populations. Previous studies in children have shown 5-year PFS rated exceeding 90%, and 10-year OS rated approaching 90% as well [18, 19, 21]. The earlier initiation of chemotherapy and higher rate of GTR in children may partially account fort he observed differences in treatment outcomes, but underlying biological differences between pediatric and adult PA are also likely contributors [11]. Remarkably, younger patients in our cohort had a significant better OS. Older patients on the other hand, with a mOS of just 26 months, appeared to fare worse than those treated with radiotherapy. However, direct comparison is limited due to cohort heterogeneity, including a higher number of prior treatment modalities in our cohort of patients [15].

While classic pediatric PA are typically driven by a single MAPK pathway alteration, adult cases often exhibit additional molecular complexity. Emerging literature suggests that alterations in ATRX, CDKN2A/B, and H3-K27M are commonly found in histologically defined PA with anaplastic features [3–8]. Reinhardt et al. defined a novel methylation class, high grade astrocytoma with piloid featured (HGAP), characterized by MAPK pathway activation, CDKN2A/B deletions, and ATRX mutations [6]. These tumors represent a distinct molecular entity with aggressive histology and clinical behavior, and are now classified as high-grade gliomas—fundamentally different from classic PA. A limitation of our study is the absence of molecular data in majority of patients, which restricted our ability to correlate genetic alterations with treatment response and prognosis.

Given the often prolonged survival of many adults with PA, the tolerability of chemotherapy is a critical consideration. In our cohort, most patients were treated with TMZ or PCV, both of which are known to be the cause of especially hematologic toxicity. Despite this, most patients completed their chemotherapy regimen without requiring discontinuation due to adverse events.

This study has several limitations that should be considered when interpreting the findings. Besides the aforementioned lack of molecular data, the retrospective design is another study limitation, which inherently introduces potential biases, including selection bias. The small sample size reflects the rarity of PA treated with chemotherapy in adults but also limits the statistical power to detect subgroup differences or treatment effects. Finally, the heterogeneity in treatment regiments, prior therapy modalities, and timing of chemotherapy initiation complicates the interpretation of outcomes and precludes definitive conclusions about the efficacy of specific agents. Despite these limitations, we believe the cohort remains representative of adults of irresectable PA, including those who have undergone prior treatments.

The findings of this study have important clinical implications. Chemotherapy appears to offer meaningful disease stabilization in adolescents and young adults (AYA). This is particularly relevant for patients who are not eligible for RT due to prior exposure or concerns about toxicity. In pediatric populations, chemotherapy is often used upfront to delay or avoid RT, aiming to prevent long-term neurocognitive decline in particularly younger children [25]. A similar strategy in adults may also improve outcomes.

Treatment paradigms are increasingly shifting toward targeted therapies, such as MEK and pan-RAF inhibitors, including second-generation agents that are also effective against BRAF::KIAA fusions [16, 17, 20, 26]. These therapies are becoming first-line options for patients with actionable molecular alterations. Nonetheless, the role of chemotherapy remains relevant for those without identifiable targets or in cases where targeted therapies are either unavailable or have failed.

Clearly defining the role of chemotherapy within this evolving treatment landscape is essential to optimize care for this rare and heterogenous patient population. To support this, we are currently developing a Dutch National Treatment Protocol for adult patients with irresectable PA. This protocol will establish clear criteria for initiating (chemo)therapy, incorporate standardized treatment and follow-up regimens, and integrate molecular outcomes in a uniformly treated patient cohort from multiple centers in analogy with the evaluation of the Dutch Society for Neuro-Oncology Treatment Protocol for Adult Medulloblastoma [27].

## Conclusion

This multicenter retrospective cohort study is the first to investigate the role of chemotherapy in adult patients with symptomatic irresectable PA. The findings indicate that chemotherapy can provide meaningful disease control with acceptable toxicity, especially in the AYA group. These results underscore the need for prospective studies of the development of standardized treatment protocols that consider early use of chemotherapy. Although treatment is increasingly shifting toward targeted therapies due to improved efficacy and tolerability of (second generation) MEK and pan-RAF inhibitors, chemotherapy may continue to have a role in patients lacking targetable alterations or following failure to targeted agents.

## Data Availability

The datasets generated during and/or analyzed during the current study are available from the corresponding author on reasonable request.

## References

[1] Palsgrove DN et al (2018) Subependymal giant cell astrocytoma-like astrocytoma: a neoplasm with a distinct phenotype and frequent neurofibromatosis type-1-association. Mod Pathol 31(12):1787–1800 29973652 10.1038/s41379-018-0103-x PMC6269209

[2] Parsons MW et al (2021) The use and efficacy of chemotherapy and radiotherapy in children and adults with pilocytic Astrocytoma. J Neurooncol 151(2):93–101 33131004 10.1007/s11060-020-03653-y

[3] Gareton A et al (2020) The histomolecular criteria established for adult anaplastic pilocytic Astrocytoma are not applicable to the pediatric population. Acta Neuropathol 139(2):287–303 31677015 10.1007/s00401-019-02088-8 PMC6989446

[4] Jones DT et al (2013) Recurrent somatic alterations of FGFR1 and NTRK2 in pilocytic Astrocytoma. Nat Genet 45(8):927–932 23817572 10.1038/ng.2682 PMC3951336

[5] Reinhardt A et al (2019) Tumors diagnosed as cerebellar glioblastoma comprise distinct molecular entities. Acta Neuropathol Commun 7(1):163 31661039 10.1186/s40478-019-0801-8 PMC6816155

[6] Reinhardt A et al (2018) Anaplastic Astrocytoma with piloid features, a novel molecular class of IDH wildtype glioma with recurrent MAPK pathway, CDKN2A/B and ATRX alterations. Acta Neuropathol 136(2):273–291 29564591 10.1007/s00401-018-1837-8

[7] Rodriguez FJ et al (2019) Alternative lengthening of telomeres, ATRX loss and H3-K27M mutations in histologically defined pilocytic Astrocytoma with anaplasia. Brain Pathol 29(1):126–140 30192422 10.1111/bpa.12646 PMC6314894

[8] Rodriguez FJ et al (2010) Anaplasia in pilocytic Astrocytoma predicts aggressive behavior. Am J Surg Pathol 34(2):147–160 20061938 10.1097/PAS.0b013e3181c75238

[9] Ostrom QT et al (2017) CBTRUS statistical report: primary brain and other central nervous system tumors diagnosed in the united States in 2010–2014. Neuro Oncol 19(suppl5):v1–v88 29117289 10.1093/neuonc/nox158 PMC5693142

[10] Salles D et al (2020) Pilocytic astrocytoma: A review of General, Clinical, and molecular characteristics. J Child Neurol 35(12):852–858 32691644 10.1177/0883073820937225

[11] Collins KL, Pollack IF (2020) Pediatric low-grade gliomas. Cancers (Basel) 12(5) 10.3390/cancers12051152 PMC7281318 32375301

[12] Koeller KK, Rushing EJ (2004) From the archives of the AFIP: pilocytic astrocytoma: radiologic-pathologic correlation. Radiographics 24(6):1693–1708 15537977 10.1148/rg.246045146

[13] Lim YJ (2022) Medical treatment of pediatric Low-Grade glioma. Brain Tumor Res Treat 10(4):221–225 36347636 10.14791/btrt.2022.0039 PMC9650116

[14] Weller M et al (2017) European association for Neuro-Oncology (EANO) guideline on the diagnosis and treatment of adult astrocytic and oligodendroglial gliomas. Lancet Oncol 18(6):e315–e329 28483413 10.1016/S1470-2045(17)30194-8

[15] Brown PD et al (2015) Adult patients with supratentorial pilocytic astrocytoma: long-term follow-up of prospective multicenter clinical trial NCCTG-867251 (Alliance). Neurooncol Pract 2(4):199–204 26640699 10.1093/nop/npv031 PMC4669035

[16] Bouffet E et al (2023) Dabrafenib plus Trametinib in pediatric glioma with BRAF V600 mutations. N Engl J Med 389(12):1108–1120 37733309 10.1056/NEJMoa2303815

[17] Fangusaro J et al (2019) Selumetinib in paediatric patients with BRAF-aberrant or neurofibromatosis type 1-associated recurrent, refractory, or progressive low-grade glioma: a multicentre, phase 2 trial. Lancet Oncol 20(7):1011–1022 31151904 10.1016/S1470-2045(19)30277-3 PMC6628202

[18] Gnekow AK et al (2012) Long-term follow-up of the multicenter, multidisciplinary treatment study HIT-LGG-1996 for low-grade glioma in children and adolescents of the German speaking society of pediatric oncology and hematology. Neuro Oncol 14(10):1265–1284 22942186 10.1093/neuonc/nos202 PMC3452343

[19] Gnekow AK et al (2019) SIOP-E-BTG and GPOH guidelines for diagnosis and treatment of children and adolescents with low grade glioma. Klin Padiatr 231(3):107–135 31108561 10.1055/a-0889-8256

[20] Kilburn LB et al (2024) The type II RAF inhibitor Tovorafenib in relapsed/refractory pediatric low-grade glioma: the phase 2 FIREFLY-1 trial. Nat Med 30(1):207–217 37978284 10.1038/s41591-023-02668-y PMC10803270

[21] Upadhyaya SA et al (2017) Brainstem Low-Grade gliomas in Children-Excellent outcomes with multimodality therapy. J Child Neurol 32(2):194–203 27810966 10.1177/0883073816675547 PMC5582383

[22] Fangusaro J et al (2020) Response assessment in paediatric low-grade glioma: recommendations from the response assessment in pediatric Neuro-Oncology (RAPNO) working group. Lancet Oncol 21(6):e305–e316 32502457 10.1016/S1470-2045(20)30064-4

[23] van den Bent MJ et al (2025) The use of minor response, volumetric assessment and growth rate kinetics as endpoints in grade 1–3 glioma clinical trials: a RANO perspective. Neuro Oncol 10.1093/neuonc/noaf173 PMC12833540 40692474

[24] Freites-Martinez A et al (2021) Using the common terminology criteria for adverse events (CTCAE - Version 5.0) to evaluate the severity of adverse events of anticancer therapies. Actas Dermosifiliogr (Engl Ed) 112(1):90–92 32891586 10.1016/j.ad.2019.05.009

[25] Patel T et al (2025) Long-term neurocognitive and behavioral outcomes in survivors of pediatric brain tumors: a systematic review. Front Neurosci 19:1587059 40520501 10.3389/fnins.2025.1587059 PMC12163034

[26] van Tilburg CM et al (2024) LOGGIC/FIREFLY-2: a phase 3, randomized trial of Tovorafenib vs. chemotherapy in pediatric and young adult patients with newly diagnosed low-grade glioma harboring an activating RAF alteration. BMC Cancer 24(1):147 38291372 10.1186/s12885-024-11820-x PMC10826080

[27] Bleeker L et al (2023) Medulloblastoma in adults: evaluation of the Dutch society for neuro-oncology treatment protocol. J Neurooncol 162(1):225–235 36920679 10.1007/s11060-023-04285-8 PMC10050065

